# Returning value to communities from the All of Us Research Program through the lens of social determinants of health and ethical, legal, and social implications

**DOI:** 10.1186/s12939-026-02758-6

**Published:** 2026-02-02

**Authors:** Malak Abu Hashish, Sophie Bronstein, Jiancheng Ye

**Affiliations:** Weill Cornell Medicine, Cornell University, New York, NY USA

**Keywords:** All of Us Program, Social determinants of health, Ethical, legal and social implications, Community-based research

## Abstract

**Objective:**

This scoping review aims to summarize the importance of community return on investment in the All of Us Research Program, particularly for enhancing the representation of underrepresented communities. It explores previous studies on social determinants of health (SDoH) and discusses the ethical, legal, and social implications (ELSI) associated with past implementations of the program. Furthermore, it discusses encountered challenges and explores strategies.

**Methods:**

We conducted a scoping review of relevant literature to examine the intersection of SDoH and ELSI within the All of Us Program. A systematic search of the literature was performed in March 2025, on PubMed/MEDLINE, EMBASE, Web of Science, Cochrane Library, and Google Scholar.

**Results:**

We identified 17 studies that focused on the intersection of the SDoH domains and ELSI. Studies showcased the importance of addressing economic, educational, and environmental factors alongside ethical considerations to enhance health equity. Despite challenges such as data completeness and participant diversity, the program’s approach to engaging with diverse communities and prioritizing ethical standards exemplifies a forward-thinking strategy in research.

**Conclusion:**

Understanding the intersection of SDoH and ELSI is crucial for the success of the All of Us Program. Integrating these factors may enhance the program’s ability to address the needs of underrepresented communities, ultimately increasing community return on investment. By continuing to address the challenges of inclusivity and data comprehensiveness, the program stands to contribute substantially to the reduction of health disparities and the advancement of healthcare for all.

**Supplementary information:**

The online version contains supplementary material available at 10.1186/s12939-026-02758-6.

## Introduction

Launched in 2016 under the National Precision Medicine Initiative by the National Institutes of Health (NIH), the All of Us Research Program represents a pioneering endeavor aimed at creating a nationally representative biobank by enrolling diverse and underrepresented participants [1]. The program’s commitment to diversity is pivotal to its stated mission of examining health conditions through various social lenses, though the realization of equitable benefits from this diversity requires ongoing attention to structural barriers and community engagement [2]. This focus not only enriches the research dataset but also has the potential to yield substantial benefits for the community, though realizing these benefits requires sustained effort and addressing systemic barriers [3, 4]. The All of Us Research Platform extends its value to communities beyond participant involvement or the return of individual results, necessitating a deeper exploration of its emphasis on social determinants of health (SDoH) and the ethical, legal, and social implications (ELSI) entailed in such a comprehensive initiative. A nuanced understanding of this intersection significantly bolsters the program’s community value. The objective of this scoping review is to review the integration of ELSI and SDoH within the All of Us Program, offering insights into the program’s strategies, challenges, and ethical considerations in fostering inclusive and equitable participation across diverse populations.

For this review, we define “underrepresented populations” as groups that have been historically excluded or marginalized in biomedical research, including but not limited to racial and ethnic minorities, individuals with disabilities, gender minorities, sexual minorities, individuals from lower socioeconomic backgrounds, rural populations, and older adults. This broad definition recognizes that underrepresentation can occur across multiple, intersecting dimensions of identity and social position.

### All of Us Research Program

At its core, the All of Us program is driven by several key objectives. One of its primary goals is to diversify biomedical research [5]. By including participants from a wide range of backgrounds—differing in race, ethnicity, age, and health status—the program hopes to create a dataset that truly reflects the diversity of the U.S. population. This inclusivity is crucial for making research findings more generalizable and equitable, addressing the gaps in knowledge that often leave underrepresented groups behind in medical advancements. Another critical objective is to advance precision medicine [6]. This approach to healthcare involves tailoring treatments and interventions to the individual, based on their genetic makeup, environment, and lifestyle [7]. The data collected through this program may help researchers understand how these factors interact and influence health and disease, with the goal of informing more personalized and effective healthcare solutions, though the pathway from data collection to improved health outcomes requires addressing multiple implementation barriers. The program also aims to foster collaborative research [6]. By providing a vast and diverse dataset to the scientific community, All of Us encourages innovation and cross-disciplinary collaboration, helping to develop new tools, technologies, and treatments that can benefit everyone. Importantly, the program views its participants as partners in research. Participants have access to their own health data and are kept informed about the program’s progress, fostering a sense of ownership and engagement in the research process.

The intended outcomes of the All of Us Research Program are as transformative as its objectives [8]. The vast and varied data collected through the program will enable the development of treatments and preventive strategies that are specifically tailored to individual needs. This personalized approach to healthcare aims to improve outcomes for patients, particularly those who have been historically underserved by the healthcare system, though the translation of research findings into clinical practice and equitable access remains an ongoing challenge. By focusing on diversity, the program also aims to reduce health disparities, ensuring that all populations can benefit from the latest medical research and advancements. Moreover, the program’s comprehensive dataset is expected to accelerate biomedical discoveries in numerous areas, including chronic disease management, cancer treatment, and mental health [9, 10].

The All of Us program is designed to be a long-term initiative. While the initial phase involves enrolling one million participants over several years, data collection and research are expected to continue for decades. This ongoing effort will provide an invaluable resource for the scientific community, supporting research that could lead to major breakthroughs in understanding and treating various health conditions. As a landmark initiative in biomedical research and the most extensive biobank project in the US, encompassing participants from all 50 states and involving a wide array of data types, the All of Us Research Program has accumulated a vast data repository, including electronic health records, physical measurements, information from wearable devices, and biospecimens [11, 12]. The recruitment strategy has been inclusive, targeting diversity across age, race/ethnicity, sexual orientation, socioeconomic status, geographic distribution, and health conditions [1]. The program’s reach also extends to genetic data, which will be used to explore how genes influence health outcomes. Participants join digitally via the program’s website or app and complete baseline health surveys, highlighting the program’s innovative and accessible approach [13].

### Social determinants of health (SDoH)

There has been a growing recognition within public health discourse of the pivotal role played by social determinants of health (SDoH) in shaping individual well-being and healthcare outcomes [14]. Acknowledging the intricate interplay between social, economic, and environmental factors becomes imperative for comprehensive healthcare delivery [15, 16].

SDoH significantly influence up to 47% of health outcomes in the US, surpassing genetic or medical conditions [17]. This underscores the substantial impact that SDoH have on people’s health, well-being, and overall quality of life [15]. In the All of Us Research Program, upon completing core surveys concerning lifestyle and overall health, participants were also given the opportunity to take optional surveys addressing areas such as healthcare access and SDoH [18]. These surveys cover five SDoH values: economic stability, education access and quality, health care access and quality, neighborhood and built environment, and social and community context [15]. Furthermore, the SDoH surveys provide researchers with a more comprehensive insight into how social determinants of health influence outcomes in various social and living settings [18].

### Ethical, legal, and social implications (ELSI)

The ELSI represent critical values that encompass the ethical, legal frameworks, and societal impacts arising from research endeavors. Serving as a guide alongside SDoH, ELSI ensures that research practices align with ethical standards and societal values [19, 20]. The framework of ELSI originated from the Human Genome Project in 1990, when researchers recognized that genomic research raised profound ethical, legal, and social questions requiring systematic attention alongside scientific inquiry [21]. This is particularly relevant to the All of Us Program’s emphasis on precision medicine, which relies heavily on genomic data. However, ELSI has evolved differently across disciplines, with varying interpretations and applications in genomics, public health, and clinical research. The implementation of ELSI principles can differ substantially based on research context, institutional frameworks, and community values, necessitating careful attention to how ELSI is operationalized in large-scale programs like All of Us.

The All of Us Program acknowledges the importance of ELSI in research, upholds three core values that guide its approach to addressing these issues. These values include inclusivity, transparency, and data accessibility. Firstly, inclusivity ensures broad participation that reflects the diversity of participants. Secondly, transparency nurtures trust by ensuring participants are fully informed about the utilization of their data. Finally, data accessibility is prioritized while maintaining stringent security measures to safeguard data privacy [19]. These ELSI are critical to ensuring the program’s efforts are ethically sound and inclusive.

Utilizing its extensive data resources, the All of Us program enriches its own initiatives while additionally providing valuable insights to the broader research community [20]. In 2019, the All of Us Research Program hosted the ELSI Priorities Workshop in Bethesda, Maryland, where ELSI professionals, NIH staff, and participant ambassadors engaged in discussions to identify key ELSI relevant to the program. The workshop topics were grounded in established ELSI principles from genomic research, including respect for persons, beneficence, justice, and privacy protection, while adapting these principles to the specific context of a large-scale precision medicine initiative. These discussions helped define the key ELSI that are explored in this review, which align with contemporary ELSI literature emphasizing inclusivity, data governance, participant engagement, and return of value to communities. The findings from this workshop have informed the focus of our study, which examines the intersection of these ELSI with the social determinants of health (SDoH) within the context of the All of Us Program [19].

### Intersectionality

A central component of the All of Us Research Program is its focus on both SDoH and the ELSI arising from such a comprehensive initiative. While the program’s aim to incorporate diverse participants is well understood, the intersection of SDoH and ELSI is equally critical. SDoH can significantly influence health outcomes, yet these determinants cannot be fully addressed without understanding the ethical, legal, and social frameworks that guide healthcare policies and interventions. Ethical considerations such as fairness, justice, and equity are vital for ensuring that health disparities are effectively reduced and that policies remain inclusive [22].

Understanding the intersection between SDoH and ELSI is key to advancing the goals of the All of Us Program. A nuanced understanding of this intersection not only strengthens the community value of the program but also enhances its ability to address systemic inequities and ensure that research findings benefit all populations. By bridging these domains, the program has the potential to foster more inclusive and equitable healthcare practices [23].

## Methods

### Search strategy

This scoping review followed the latest version of the preferred reporting items for systematic reviews and meta-analyses for scoping review (PRISMA-ScR) guideline for the whole review process [24]. The search was conducted across multiple databases, including PubMed/MEDLINE, EMBASE, Web of Science, Cochrane Library, and Google Scholar, covering publications up to March 2025. The search strategy was designed to capture a broad spectrum of research articles, reviews, reports, and policy documents relevant to the All of Us Research Program, with a particular emphasis on Social Determinants of Health (SDoH) and Ethical, Legal, and Social Implications (ELSI). Details of the search strategy are demonstrated in Supplemental Table 1.

Specific terms and keywords were identified and combined using Boolean operators (AND, OR) to maximize search efficiency and inclusivity. These included “All of Us Research Program,” “social determinants of health,” “SDoH,” “Ethical, Legal, and Social Implications,” “ELSI,” “community engagement,” “diversity in research,” “underrepresented populations,” “biobanking,” “genomic data,” “data sharing,” “participant return of results,” and “health equity.” Both controlled vocabulary terms (e.g., MeSH terms) and free-text terms were used to ensure comprehensive coverage. The search was not limited by publication type, but was restricted to English-language articles to ensure relevance to the target audience as well as due to practical limitations related to translation resources. Additionally, subdomains of SDoH and ELSI, such as “economic stability,” “education quality,” and “healthcare access,” were included to capture a wider range of literature and ensure the search’s completeness.

### Selection criteria and process

The selection process was rigorous and methodologically sound to ensure the inclusion of high-quality, relevant studies. The inclusion criteria were defined a priori and focused on English-language articles that addressed at least one of the following: the All of Us Research Program, Social Determinants of Health (SDoH), or Ethical, Legal, and Social Implications (ELSI). Articles that discussed the impact of the All of Us Program on communities, particularly those from underrepresented populations, and those that explored issues of diversity, equity, and inclusion in the context of SDoH or ELSI were prioritized.

The selection process was conducted in several stages. Before the screening process began, a standardized screening form was developed and pilot-tested by the review team. The quality of the included studies was assessed using adapted criteria from the Mixed Methods Appraisal Tool (MMAT) [25] and included evaluation of: (1) clarity of research objectives, (2) appropriateness of study design, (3) sample size adequacy and representativeness, (4) validity and reliability of data collection methods, (5) appropriateness of analysis methods, and (6) robustness and generalizability of findings. Studies were rated as high, moderate, or low quality, with all included studies meeting at least moderate quality standards. Two independent reviewers conducted this screening to minimize bias and ensure consistency. For studies where inclusion or exclusion could not be confidently determined based on the title and abstract, the full text was retrieved for further review. Both reviewers independently assessed the full text to confirm eligibility based on the predefined criteria. In cases where the two reviewers disagreed on the inclusion of a study, a third reviewer was consulted. The third reviewer independently reviewed the study and discussed the findings with the initial reviewers until a consensus was reached. If necessary, the discrepancies were resolved through team discussions to ensure that the selection process was transparent and reproducible. Once the final set of studies was selected, data extraction was carried out using a standardized extraction form. This form captured key information such as study objectives, methods, findings, and relevance to the All of Us Program, SDoH, and ELSI. The quality of the included studies was assessed based on criteria such as study design, sample size, and the robustness of the findings.

### Quality assessment

In evaluating the studies, several key dimensions were considered to ensure a comprehensive and rigorous review. First, coverage of SDoH was assessed across five domains: economic stability, education access and quality, healthcare access and quality, neighborhood and built environment, and social and community context. Each study was scored based on how thoroughly it addressed these interconnected factors that shape individual and community well-being. In parallel, ELSI coverage was evaluated through three core values: inclusivity, transparency, and data accessibility. These principles reflect the broader commitment to equity and responsible data stewardship in health-related research.

To gauge the reliability of the data presented, a detailed quality assessment was conducted. This included examining the adequacy and representativeness of sample sizes, the rigor of data collection methods—such as the use of validated instruments and standardized protocols—and the potential for bias, whether through selection, recall, or measurement. Completeness of the data was also a critical factor in this evaluation. Finally, each study received an overall quality rating. Studies rated as “High” demonstrated rigorous methodology, employed large and representative samples or comprehensive analyses, used validated measures, and applied appropriate statistical methods with minimal bias. Those rated as “Moderate” showed generally sound methodology but had some limitations, such as smaller sample sizes, reliance on self-reported data, or potential selection bias, though their analyses remained appropriate. Importantly, studies with significant methodological flaws were excluded from the final review, ensuring that only robust and credible evidence was considered.

### Data synthesis

The selected studies were synthesized qualitatively using thematic analysis following Braun and Clarke’s framework [26]. The synthesis process involved: (1) familiarization with the data through repeated reading of all included studies, (2) generating initial codes related to SDoH domains and ELSI values, (3) identifying and organizing codes into potential themes, (4) reviewing and refining themes to ensure they accurately represented the data, and (5) defining and naming final themes. Two independent reviewers conducted the thematic analysis, with discrepancies resolved through discussion until consensus was reached. The synthesis focused on identifying common themes, gaps, and implications related to the All of Us Research Program, with particular attention to how the program addresses (or fails to address) SDoH and the ELSI of its operations. The findings were critically analyzed in the context of existing literature and ongoing debates within the fields of public health, ethics, and precision medicine.

## Results

**Fig. 1:**
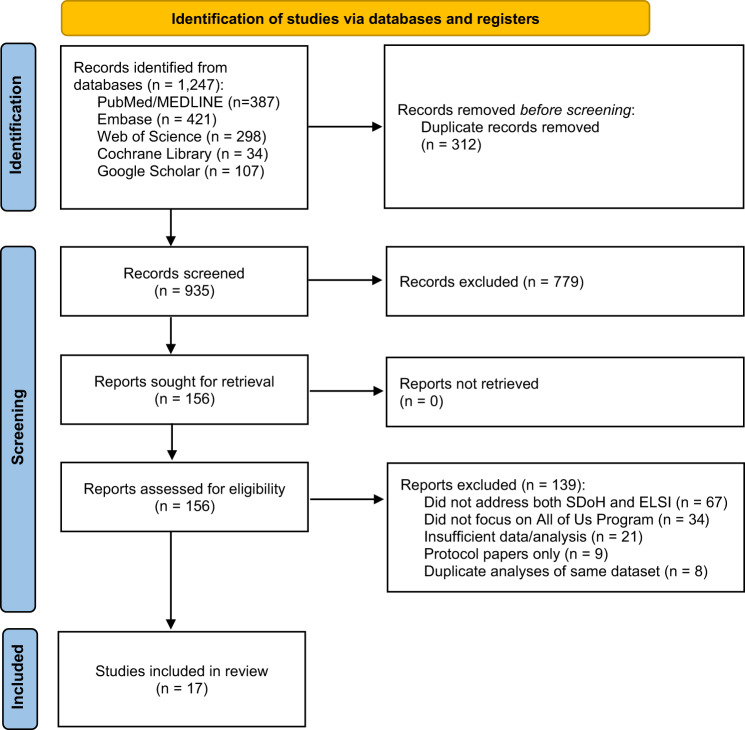
PRISMA flow diagram for study selection process

**Fig. 2:**
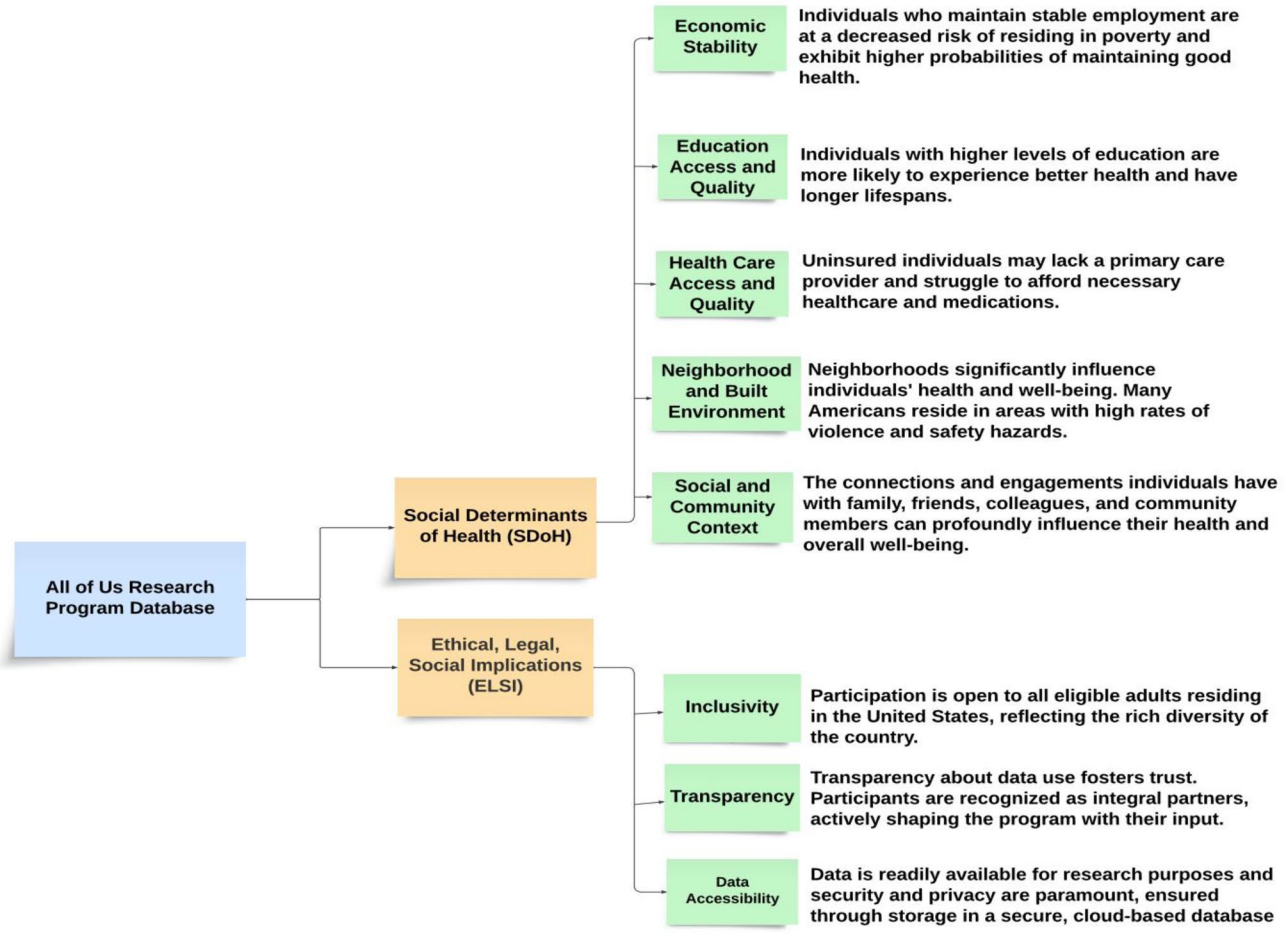
The framework of the intersections of social determinants of health (SDoH) domains and Economic, legal, and social implications (ELSI) values for All of Us Program

Figure 1 illustrates the PRISMA-ScR flow diagram of the included studies in the review. The initial database search yielded 1247 articles. After removing duplicates (*n* = 312), 935 articles underwent title and abstract screening. Of these, 156 articles were selected for full-text review. Following full-text assessment, 17 studies met all inclusion criteria and were included in the final analysis. We proposed a framework for this study in Fig. 2 that demonstrates the intersection of SDoH domains and ELSI. This framework visually depicts the comprehensive breakdown of SDoH and ELSI within the All of Us Program, showcasing their respective domains and core values. Table 1 demonstrated the selected studies that examined the intersection of SDoH and ELSI within the All of Us Research Program. Collectively, these studies demonstrated the multifaceted nature of SDoH and ELSI and highlighted their significant impact on health outcomes and healthcare access, especially among underrepresented populations: populations that have been historically excluded or marginalized in research.

**Table 1:** Summary of the selected studies

Study	SDoH insights	ELSI insights	Lessons/implications
Bhavnani, 2023 [27]	Identified distinct SDoH subtypes that contributed to delayed access to medical care, with a focus on risk stratification.	Included a demographically diverse cohort, particularly adults aged ≥65 years, within the All of Us Program.	Demonstrated the importance of recognizing SDoH profiles that act as barriers to timely care, with diversity in the All of Us Program providing a strong platform for these analyses.
Felker, 2024 [17]	Reported that SDoH accounted for up to 47% of overall health outcomes, with emphasis on their influence in the presentation and diagnosis of neurofibromin 1 (NF1)-related conditions.	Analyzed NF1 genomic variants among 182,492 participants, linking molecular data with social context.	Showed that integrating SDoH data with highly penetrant genetic disorders can provide nuanced insights into disease expression and diagnosis.
Fisher, 2020 [28]	Highlighted persistent underrepresentation of racial and ethnic minority populations in biomedical research.	Explored minority groups’ perspectives and attitudes, which informed recruitment and design strategies within the All of Us Program.	Future studies should incorporate the socio-ecological framework to address structural and individual-level barriers to participation.
Friedman, 2017 [29]	Outlined a comprehensive strategy to advance health equity through addressing upstream SDoH factors.	Emphasized the role of legal and policy instruments in shaping equitable health interventions within and beyond healthcare systems.	Illustrated the critical role of structural and legal mechanisms in overcoming barriers to health equity.
Hammack, 2019 [30]	Recognized the value of large-scale, diverse data collection across race, ethnicity, age, and sex to reflect U.S. demographics.	Consulted nationally recognized experts in confidentiality, genomics, and ELSI to address concerns about data security.	Stressed the need for proactive preventive measures to safeguard participant privacy and genomic data integrity.
Huang, 2023 [18]	Used All of Us data to identify SDoH-related barriers among patients with diabetic retinopathy (DR), including economic and geographic obstacles.	Highlighted the importance of diversifying the ophthalmology workforce, as patients often prefer culturally or demographically concordant physicians.	Demonstrated how SDoH-related barriers delay care, underscoring the need to prioritize workforce diversity and health equity.
Kunnath, 2024 [3]	Characterized socio-demographic profiles of All of Us participants and examined predictive values of SDoH for chronic disease risk.	Recognized the All of Us Program’s role in advancing equitable health research through demographic inclusivity.	Identifying high-risk sociodemographic groups enables tailored interventions and supports precision health equity.
Lee, 2022 [4]	Implemented SDoH-specific surveys for participants with eye conditions, enriching datasets for ophthalmology research.	Showed that All of Us demographic distribution closely mirrored national diversity, with strong representation of Black and Hispanic participants.	Emphasized the need to expand SDoH data collection systematically to support comprehensive and representative analyses.
Lewis, 2023 [31]	Evaluated underrepresentation of blind and deaf participants in the program, identifying disability-related disparities.	Suggested that a person-centered approach requires intentional inclusion of individuals with disabilities, who experience disproportionate health inequities.	Highlighted the universal impact of disability, calling for structured mechanisms to ensure equitable representation in research.
Luo, 2022 [11]	Examined the association between housing type and COVID-19 infection risk, showing elevated transmission in communal living environments.	Noted the program’s emphasis on transparency and inclusion, with de-identified data shared through a secure cloud infrastructure.	Demonstrated the value of integrating environmental SDoH (housing) with health outcomes, especially in infectious disease contexts.
Lyles, 2018 [32]	Framed social determinants as fundamental drivers of health requiring systemic integration into research.	Identified the need for improved infrastructure, interoperable data systems, and governance frameworks to build participant trust.	Stressed future directions for precision population health, requiring robust SDoH integration and governance mechanisms.
Ma, 2022 [33]	Used “Basics” and COPE surveys to analyze how SDoH influenced COVID-related behaviors among older adults.	Noted NIHs commitment to including underrepresented populations, such as women and racial/ethnic minorities, within the All of Us Program.	Recommended continuous updates to SDoH variables to capture emerging challenges and evolving contexts.
Mensah, 2019 [1]	Explored modifiable SDoH-related risk factors contributing to elevated cardiovascular disease burden among minority populations.	Addressed ethical imperatives of conducting genomic research in vulnerable groups and emphasized the role of community trust.	Pointed to risks of exacerbating disparities if genomic research lacks adequate diversity and inclusion.
Rasooly, 2023 [13]	Investigated the interplay between family history and SDoH in shaping risks for obesity, diabetes, and cardiovascular conditions.	Reported identification of 225 family history associations across socioeconomically and racially diverse cohorts.	Demonstrated how diverse participation enhances the ability to generalize family history associations across demographic groups.
Ravindranath, 2023 [34]	Analyzed links between diabetic retinopathy screening uptake and SDoH factors such as food insecurity, housing instability, and mental health.	Highlighted the role of cultural concordance and workforce diversity in mitigating barriers to care.	Showed that SDoH and patient–provider concordance strongly influence care utilization and health outcomes.
Sankar, 2017 [6]	Identified the intersection of SDoH and ELSI concerns in perpetuating health disparities.	Recommended that the All of Us Program establish a formal ELSI research initiative to track and address emerging issues.	Underlined the necessity of continuous ELSI research to support equitable implementation of precision medicine programs.
Schulkey, 2023 [35]	Used the COPE survey to capture COVID-19-related SDoH factors across diverse U.S. populations.	Detailed the All of Us Program’s processes for de-identification, encryption, and secure data sharing via the Research Hub.	Recommended expanding SDoH variables, particularly those addressing mental health, to strengthen future analyses.

Table 2 presents the methodological quality assessment of all 17 included studies. Using adapted criteria from the Mixed Methods Appraisal Tool (MMAT), we evaluated each study across multiple dimensions including study design appropriateness, sample characteristics, data quality, and analysis methods. The majority of included studies (*n* = 11, 64.7%) were rated as high quality, demonstrating rigorous methodology, adequate sample sizes, validated measurement instruments, and appropriate analytical approaches. The remaining six studies (35.3%) were rated as moderate quality, typically due to limitations such as smaller sample sizes, reliance on self-reported data with potential recall bias, or theoretical rather than empirical approaches. Coverage of SDoH domains was comprehensive across included studies, with a mean of 4.1 out of 5 domains addressed per study (range: 2–5). Ten studies (58.8%) addressed all five SDoH domains. ELSI coverage averaged 2.4 out of 3 core values per study (range: 2–3), with six studies (35.3%) addressing all three core ELSI values (inclusivity, transparency, and data accessibility). No studies were rated as low quality; such studies were excluded during the full-text screening process as they did not meet our predetermined quality thresholds. The quality assessment revealed several patterns. Quantitative studies with large All of Us datasets consistently achieved high quality ratings due to sample size, diversity, and use of validated instruments. Studies that were primarily conceptual or review-based received moderate ratings when they provided comprehensive frameworks but lacked empirical data, though those offering rigorous legal or ethical analysis achieved high ratings. Studies addressing disability populations or specific disease conditions demonstrated particular strength in ELSI considerations, especially regarding inclusivity and equity.

**Table 2:** Methodological quality assessment of included studies

Study	Study Design	Sample Size	Data Source	SDoH Coverage*	ELSI Coverage†	Data Quality‡	Analysis Method	Overall Quality§
Bhavnani, 2023 [27]	Cross-sectional	16,795 participants ≥65 years	All of Us surveys and EHR	4/5 (Economic, Education, Healthcare, Social context)	2/3 (Inclusivity, Transparency)	High - standardized instruments	Cluster analysis, logistic regression	High
Felker, 2024 [17]	Cross-sectional genomic analysis	182,492 participants	Genomic, phenotypic, survey data	5/5 (All domains)	2/3 (Inclusivity, Data accessibility)	High - validated genomic data	Genetic variant analysis, multivariable models	High
Fisher, 2020 [28]	Systematic review	52 studies reviewed	Qualitative/quantitative literature	3/5 (Economic, Education, Social context)	3/3 (All core values)	Moderate - heterogeneous studies	Thematic synthesis	Moderate
Friedman, 2017 [29]	Conceptual framework	N/A (theoretical)	Literature review	5/5 (All domains)	3/3 (All core values)	High - comprehensive framework	Narrative synthesis, legal analysis	High
Hammack, 2019 [30]	Qualitative interview study	25 ELSI experts	Key informant interviews	2/5 (Healthcare, Social context)	3/3 (All core values)	High - expert perspectives	Thematic analysis	High
Huang, 2023 [18]	Cross-sectional	5,010 participants with DR	Survey data, EHR	4/5 (Economic, Healthcare, Social context, Neighborhood)	2/3 (Inclusivity, Transparency)	High - validated outcomes	Multivariable logistic regression	High
Kunnath, 2024 [3]	Cross-sectional descriptive	329,070 participants	Survey data	5/5 (All domains)	2/3 (Inclusivity, Data accessibility)	High - large representative sample	Predictive modeling, RF analysis	High
Lee, 2022 [4]	Cross-sectional	42,165 participants with eye conditions	Survey data, EHR	5/5 (All domains)	2/3 (Inclusivity, Data accessibility)	High - comprehensive SDoH surveys	Descriptive statistics, chi-square tests	High
Lewis, 2023 [31]	Cross-sectional comparative	413,457 participants	Survey data	3/5 (Healthcare, Social context, Economic)	3/3 (All core values)	High - disability-specific analysis	Comparative statistics, regression	High
Luo, 2022 [11]	Case-control	101,709 participants	COPE survey, demographics	4/5 (Economic, Neighborhood, Healthcare, Social context)	2/3 (Transparency, Data accessibility)	Moderate - self-reported COVID status	Logistic regression, propensity matching	Moderate
Lyles, 2018 [32]	Conceptual/perspective	N/A (commentary)	Literature synthesis	5/5 (All domains)	3/3 (All core values)	Moderate - expert opinion	Narrative synthesis	Moderate
Ma, 2022 [33]	Cross-sectional	7,443 older adults	COPE survey, Basics survey	4/5 (Economic, Healthcare, Social context, Neighborhood)	2/3 (Inclusivity, Transparency)	Moderate - convenience sample	Decision tree modeling	Moderate
Mensah, 2019 [1]	Narrative review	N/A (review article)	Literature synthesis	4/5 (Economic, Education, Healthcare, Social context)	2/3 (Inclusivity, Transparency)	Moderate - selective review	Narrative synthesis	Moderate
Rasooly, 2023 [13]	Cross-sectional	245,394 participants	Survey data, family history	3/5 (Economic, Social context, Healthcare)	2/3 (Inclusivity, Data accessibility)	Moderate - self-reported history, recall bias	Logistic regression, stratified analysis	Moderate
Ravindranath, 2023 [34]	Cross-sectional	4,977 participants with diabetes	Survey data, screening records	4/5 (Economic, Healthcare, Social context, Neighborhood)	2/3 (Inclusivity, Transparency)	High - validated screening measures	Multivariable regression, mediation analysis	High
Sankar, 2017 [6]	Commentary/framework	N/A (policy paper)	Literature review	3/5 (Economic, Healthcare, Social context)	3/3 (All core values)	High - comprehensive ELSI framework	Legal and ethical analysis	High
Schulkey, 2023 [35]	Cross-sectional	315,686 participants	COPE survey	5/5 (All domains)	3/3 (All core values)	High - large, diverse sample	Descriptive statistics, survey validation	High

* SDoH Coverage (scored out of 5 domains): 1) Economic Stability, 2) Education Access and Quality, 3) Healthcare Access and Quality, 4) Neighborhood and Built Environment, and 5) Social and Community Context

† ELSI Coverage (scored out of 3 core values)

Inclusivity

Transparency

Data Accessibility

‡ Data Quality Assessment based on:

Sample size adequacy and representativeness

Data collection methods (validatedinstruments, standardized protocols)

Potential for bias (selection,recall, measurement) Completenessof data

Completenessof data

§ Overall Quality Rating:

High: Rigorous methodology, large representative sample or comprehensive analysis,validated measures, appropriate statistical methods, minimal bias

Moderate: Adequate methodology with some limitations (e.g., smaller sample, self-reporteddata, potential selection bias), appropriate analysis

Low: Significant methodological limitations (Note: No studies rated as low qualitywere included in final review)

A key theme that emerged from these studies was the intricate interplay between genetic predispositions and social factors in shaping health outcomes. While this intersection is expected given the program’s focus on both genomic data and SDoH, the selected studies provided nuanced empirical evidence of how these factors interact in specific disease contexts and demonstrated methodological approaches for integrating genetic and social data. For example, Felker’s study utilized genomic, phenotypic, and survey data from 182,492 participants in the All of Us Research Program to explore factors influencing the presentation and diagnosis of neurofibromatosis type 1 (NF1) [17]. This study highlighted the importance of considering both genetic and social determinants when addressing health disparities, particularly in diverse populations. Similarly, Huang et al.‘s cross-sectional study on Diabetic Retinopathy (DR) among All of Us participants emphasized how lower socioeconomic status (SES) was associated with a higher prevalence and severity of DR [18]. Their analysis of the SDoH surveys revealed how various SDoH factors, such as access to healthcare and support systems, influence the experiences of individuals with DR. The study also underscored the importance of diversifying the ophthalmology workforce to better address the needs of diverse populations. Additionally, their analysis of the SDoH survey identified seven key questions that shed light on participants’ experiences within healthcare settings [18]. These questions explored support system access, healthcare interactions, treatment perceptions, and communication during appointments [18]. Ravindranath et al.‘s research aimed to explore the relationship between patient-reported factors for diabetic retinopathy screening and SDoH outcomes. Their findings revealed that individuals from diverse populations experiencing insecurities such as food or housing were less likely to seek eye care compared to those without such insecurities [34].

Some studies emphasized the importance of demographic factors in predicting health outcomes. Kunnath et al. conducted a cross-sectional analysis using deidentified survey data from the All of Us Program, which revealed the relative importance of sociodemographic factors in predicting chronic diseases [3]. Their findings underscored the need for targeted interventions that address health disparities among diverse populations. Their findings illuminate the interaction between demographic characteristics and health outcomes, emphasizing the necessity of targeted interventions to address disparities among diverse populations [3]. Lee et al. further explored the coverage of SDoH data within the All of Us Program, focusing on demographic and social variables recommended by the Institute of Medicine (IOM) [4]. Their comprehensive examination of various social determinants offers a thorough understanding of the factors contributing to health disparities among diverse populations. The study revealed higher participation rates among Black and Hispanic individuals, highlighting the bidirectional relationship between SDoH and ophthalmic conditions. This emphasized how low visual acuity can act as a risk factor for adverse social outcomes, worsened mental health, and poverty [4]. The importance of inclusivity in biomedical research was also a recurring theme [36]. The research by Lewis et al. illuminated the distinct health challenges faced by blind and deaf adults participating in the All of Us Research Program [31]. By spotlighting this underrepresented group, the study underscores the importance of inclusivity in biomedical research and the need to address the requirements of diverse and underrepresented communities [31].

A few studies also addressed the impact of the COVID-19 pandemic on underrepresented populations. Luo et al., investigated the relationship between housing types and COVID-19 infection rates using data from the All of Us Program COPE survey conducted in 2021; the aim of this survey was to comprehend the impact of COVID-19 on the daily lives and health of underrepresented participants, particularly their mental well-being [37]. It covered a range of topics, including social distancing experiences, self-reported COVID-19 status, well-being, participant demographics, mental health, COVID-19-related socioeconomic changes, and physical activity [11, 35, 38]. Similarly, a parallel study conducted by Ma et al., suggested that SDoH have been linked to worsening COVID-19 outcomes along with heightened treatment disparities, especially among the elderly [33]. Moreover, Rasooly et al.‘s study reveals 225 family history links to obesity, diabetes, heart, and blood (ODHB) conditions among racially and economically diverse US adults in the All of Us Program [13].

Several studies also highlighted the role of legal and ethical frameworks in addressing health disparities and promoting equity. Friedman & Gostin’s study emphasized the multifaceted nature of SDoH and ELSI legal instruments; These instruments operate across various levels, including biases among healthcare professionals, community-level disparities, and political influences [29]. This aligns with the core ELSI of inclusivity, transparency, and data accessibility. Lastly, a study implemented Mobile health (mHealth)in conjunction with geospatial sensors to analyze crowdsensing data [39, 40]. This assisted participants with tinnitus and health-conscious individuals to steer clear of locations with harmful sounds [39] mHealth utilizes mobile computing and communication technologies in healthcare and public health, is rapidly growing within the realm of digital health [41, 42].

Transparency and trust emerged as crucial elements throughout the studies, emphasizing the importance of building trust among diverse populations and ensuring transparency in research endeavors. The importance of workforce diversity and cultural competency was consistently highlighted, emphasizing the necessity for healthcare practitioners to reflect the diversity of the populations that they serve [34].

Several studies explicitly addressed the ELSI of the All of Us Research Program, although the depth and focus varied. For instance, Lewis et al. emphasized the importance of inclusivity within biomedical research, a core ELSI, by highlighting the distinct health challenges faced by blind and deaf participants [31]. This study underscored the necessity for research practices that are sensitive to the needs of underrepresented groups, aligning with the ELSI of equity and respect for diverse populations. Similarly, Friedman & Gostin explored the legal dimensions of ELSI, particularly focusing on how legal frameworks can either mitigate or exacerbate health disparities [29]. However, not all studies in the review gave ELSI the same level of attention, which may reflect a broader challenge in consistently integrating ELSI into research practices [29]. The connection between SDoH and ELSI was evident in several studies, particularly those that examined how social factors like socioeconomic status, education, and access to healthcare influence health outcomes. These studies recognized that addressing SDoH was inherently an ELSI issue, as it pertains to ensuring that research benefits are equitably distributed and that vulnerable populations are adequately protected. However, the literature also highlighted gaps where unaddressed ELSI challenges could potentially limit the generalizability of findings. For example, studies that did not fully consider the ethical implications of data privacy or informed consent in communities with low literacy rates may have overlooked key aspects of participant protection, thereby affecting the validity and applicability of their conclusions. These studies often suggested that the All of Us Research Program needs to strengthen its focus on ELSI, particularly by developing strategies to better engage underrepresented populations and by ensuring that SDoH considerations are fully integrated into the ethical framework guiding research practices [19, 20].

## Discussion

This scoping review shed light on various aspects of community health empowerment within the All of Us Program, emphasizing its dedication to equitable research among diverse and underrepresented populations. We analyzed the findings and implications of the selected studies, and examined the intersections of SDoH and ELSI. The intersection is a dynamic space where the broader social context of health intersects with the ethical responsibilities of research. Within this intersection, the SDoH domains inform the lived experiences of individuals and communities, shaping their health outcomes and access to care [43]. ELSI, on the other hand, provide a framework to ensure that research practices are aligned with principles of fairness, respect, and justice.

Studies that examine SDoH without an ethical framework may overlook how power dynamics, historical injustices, and structural inequities shape both health determinants and research participation. Conversely, ELSI discussions that do not adequately incorporate SDoH analysis may propose ethical solutions that are theoretically sound but practically insufficient for addressing the lived experiences of marginalized communities. This intersection is not merely additive but transformative, requiring researchers to consider how social context shapes ethical obligations and how ethical principles must be operationalized differently across diverse social positions. In the All of Us Program, this intersection is critically important for several reasons. First, it demands that the program not only collects and analyzes data related to SDoH but also does so in a way that is ethically sound and legally compliant. For example, when examining the impact of neighborhood environments on health, researchers must navigate privacy concerns and ensure that data usage does not reinforce existing inequities. Second, this intersection challenges the program to consider the social implications of its findings—how they might influence public policy or healthcare practices, and whether they contribute to reducing or exacerbating health disparities. Finally, the intersection requires commitment to community engagement and transparency, ensuring that participants, especially those from marginalized or underrepresented groups are not only protected but also meaningfully involved in the research process.

### Empowering community health

The All of Us Research Program aims to contribute to community health empowerment, particularly among underrepresented and marginalized populations, though significant challenges remain in translating research participation into tangible health benefits. While the program has implemented inclusive recruitment strategies and community engagement mechanisms, the pathway from data collection to community empowerment requires addressing longstanding structural barriers that the program alone cannot resolve. Historically, these communities have faced systemic barriers to healthcare access and research participation, stemming from deep-rooted mistrust due to past injustices and exploitation in healthcare research. This lack of trust remains a significant obstacle to minority participation in research projects [28], highlighting the importance of acknowledging past injustices and building trust to encourage participation in research projects within diverse communities [28].

Efforts to establish community value and enhance health outcomes encounter challenges in accessing, engaging, and retaining participants from socially disadvantaged groups [44]. While the concept of diversity in research is expanding, precision in its application requires careful attention [45]. Boosting the representation of minorities in studies not only facilitates ethnicity-specific analyses and data presentation but also addresses disparities in healthcare and research benefits across underrepresented groups [46, 47].

When examining studies that focus solely on either SDoH or ELSI, it is crucial to recognize the consequences of neglecting the intersection between these two domains. This intersection represents the central analytical contribution of our review. Research that isolates one factor risks overlooking how the broader social context influences ethical concerns or how ethical considerations shape the interpretation of social determinants. For instance, failing to integrate SDoH may result in incomplete ethical discussions around fairness and justice, missing opportunities to address systemic inequities that determine who benefits from research and who bears its burdens. A study might appropriately address informed consent procedures but fail to recognize how educational disparities, language barriers, or historical trauma influence whether consent is truly informed or voluntary across different communities. Conversely, focusing exclusively on SDoH without an ethical framework could inadvertently reinforce existing disparities by neglecting the nuanced needs and concerns of vulnerable populations, such as privacy risks that may be heightened for communities with histories of surveillance or discrimination. The intersection of SDoH and ELSI is therefore essential to fully understanding and addressing the challenges and opportunities within initiatives like the All of Us Program, ultimately enhancing its ability to engage diverse communities and reduce health disparities rather than simply documenting them [23].

The All of Us Research Program contributes to community empowerment and well-being through community outreach and involvement. By fostering trust and bridging gaps between marginalized communities and healthcare institutions, the program not only advances scientific understanding but also contributes to the empowerment and well-being of diverse communities. Strategies aimed at enhancing minority participation in health research should prioritize improving access to research opportunities for these communities [48]. Efforts to improve access can include actively inviting participation, ensuring accessible study sites, and addressing barriers such as childcare needs or travel expenses reimbursement. Moreover, as the program expands, it should explore ancestry-matched controls and consider cultural, social, and environmental factors in its research design to ensure inclusivity and relevance to diverse populations.

By actively considering and addressing these factors, the All of Us Program can foster greater inclusivity, engagement, and trust within the diverse communities it serves. This, in turn, will enhance research outcomes and benefit the communities, ultimately contributing to the program’s ability to address health disparities and promote equitable access to healthcare.

### Barriers and challenges

One significant challenge encountered by the All of Us Research Program is the underrepresentation of certain demographic groups in its surveys. For instance, the SDoH survey participants primarily consisted of older individuals and males. This skewed representation limits the generalizability and applicability of the data to younger or female populations [18]. The historical underrepresentation of women in healthcare research is particularly concerning, as many studies have either excluded women entirely or failed to report or analyze gender-specific data [49]. Addressing this imbalance is critical to ensuring that research findings are inclusive and applicable to all segments of the population.

The division of SDoH variables into mandatory and optional surveys has led to incomplete data, which can introduce selection bias and compromise the external validity of the findings [34]. The optional nature of some surveys may deter participants from completing them, resulting in gaps in the data. This incomplete data could be attributed to the lengthy and time-consuming nature of the surveys, which may discourage participation and thoroughness. Streamlining the survey process and incentivizing complete participation are essential steps to mitigate this issue [4].

Additionally, certain SDoH variables lack comprehensive coverage within the All of Us Program. For example, gender identity as an SDoH has been underexplored and unrecognized, limiting the ability to fully understand its impact on health outcomes [5]. Regularly updating and fact-checking SDoH data is essential to ensure its relevance and accuracy. Expanding the scope of SDoH variables to include a broader range of factors will provide a more holistic understanding of the social determinants influencing health.

The inclusion of self-reported family and personal medical history raises concerns about measurement error influenced by baseline sociodemographic traits and recall bias [3, 13]. Participants’ recollections may be inaccurate or biased, affecting the reliability of the data. Adapting survey methodology to reduce these biases is crucial. This could involve using more objective measures, corroborating self-reported data with medical records, or employing more precise questioning techniques.

The historical mistreatment and exploitation of racial and ethnic minority groups in medicine and research have created a deep-rooted mistrust in healthcare providers and systems [50]. This mistrust is frequently identified as a major obstacle to minority populations participating in research [51]. Efforts to build trust must acknowledge past injustices and demonstrate a genuine commitment to ethical and inclusive research practices [52]. Transparent communication about the goals, benefits, and risks of participation can help build trust among potential participants. Ensuring diversity within the scientific team and staff conducting the research is crucial [53]. A diverse research workforce is more likely to understand and address the needs and concerns of underrepresented communities. Efforts should be made to recruit and retain researchers from diverse backgrounds and to provide training on cultural competency and inclusivity [54].

Technological barriers also pose significant challenges. While the All of Us Program utilizes innovative digital platforms for participant recruitment and data collection, disparities in access to technology can limit participation from certain groups [55]. Efforts to bridge the digital divide, such as providing access to necessary technology and offering alternative methods of participation for those without digital access, are essential [56].

Concerns about privacy and data security are prevalent among potential participants, particularly those from marginalized communities who may have heightened fears of misuse of their information [57]. The program must implement and communicate robust privacy and security measures to protect participant data. Ensuring transparency about how data will be used and safeguarding it against breaches are critical for building trust [58]. Logistical and resource constraints can hinder the program’s ability to reach and engage diverse populations. Addressing barriers such as travel costs, time commitments, and accessibility of research sites is important [59]. Providing resources such as transportation assistance, flexible scheduling, and remote participation options can help mitigate these challenges [60].

### Advancing equity, inclusion, and community benefits through ELSI

Multiple studies have emphasized the ELSI associated with the All of Us Program [61, 62]. The effectiveness of such research hinges on grasping the intricate network of laws, regulations, policies, and procedures to clarify risk explanations, assess participant protection levels, and enhance privacy and confidentiality safeguards [30]. The creators of the program have taken proactive steps to address specific ELSI, such as implementing stringent privacy measures and comprehensive participant engagement strategies [6]. This emphasizes the significance of using ELSI research findings to shape continuous governance, policies, and practices within the All of Us Program.

The All of Us Program recognizes the importance of addressing historical injustices in biomedical research. Communities of color and other marginalized groups have historically faced exploitation and unethical treatment. These past abuses have created a legacy of mistrust towards healthcare research and institutions. By openly acknowledging this history, the program takes a significant step towards rebuilding trust. It prioritizes transparency, ensuring that participants are fully informed about how their data will be used, thereby fostering a sense of ownership and engagement. The program has taken proactive steps to address specific ELSI. Stringent privacy measures are in place to protect participant data, including secure data storage, de-identification of personal information, and robust consent processes. These measures are designed to uphold the highest ethical standards and legal requirements, ensuring that participants feel confident in the security of their personal information. This approach not only builds trust but also aligns with the program’s core values of inclusivity and transparency [5].

The All of Us Program should also acknowledge and address ongoing concerns about its own practices with specific communities. Tribal nations, for instance, have raised important questions about data sovereignty, the adequacy of consultation processes, and whether the program sufficiently respects tribal authority over research involving their members [63]. Despite extensive listening sessions conducted with tribal communities, concerns persist about the extent to which tribal feedback has meaningfully shaped program policies, particularly regarding data governance, benefit-sharing, and the right to withdraw data [64]. These ongoing tensions highlight that acknowledging past injustices, while necessary, is insufficient without demonstrating genuine commitment to power-sharing and community self-determination in research governance.

Feedback from participants plays a crucial role in the program’s ELSI. The All of Us Program has established multiple channels for collecting participant feedback, ensuring that their voices are heard and their concerns addressed. Participant ambassadors, who serve as liaisons between the program and the community, provide valuable insights and help identify areas for improvement. This feedback mechanism fosters an open dialogue that enhances transparency and trust. By actively involving participants in research prioritization, data analysis strategies, and knowledge dissemination efforts, the program ensures that it remains responsive to the needs and concerns of its diverse participant base [19]. Inclusivity is also a key pillar of the All of Us Program. The program strives to reflect the diversity of its participants within its staffing and research teams. This inclusivity is crucial for conducting culturally competent research that is relevant and respectful of different communities. Cultural competency involves understanding and addressing the needs, values, and perspectives of diverse populations, thereby enhancing the relevance and impact of the research. By ensuring that its scientific team and staff are diverse, the program builds trust and improves the quality of its research outcomes [32].

Community empowerment is another significant aspect of the All of Us Program’s approach to ELSI. The program involves community members in the research process and provides them with access to research findings and resources that can help them advocate for better health outcomes and policies. This involvement empowers communities to take an active role in addressing systemic issues that impact their well-being. By focusing on SDoH and ELSI, the program aims to develop interventions and policies that reduce health disparities and promote equitable access to healthcare for all populations. Continuous improvement is a cornerstone of the All of Us Program’s ELSI strategy. The program uses ELSI research findings to inform its governance, policies, and practices, ensuring that it remains adaptive and responsive to new challenges and opportunities in biomedical research. This iterative process involves regularly reviewing and updating research practices, consent processes, and engagement strategies to align with evolving ethical standards and participant expectations. By committing to continuous improvement, the program ensures that it can effectively address the complex health needs of diverse populations.

### Strategies and recommendations

Ensuring diversity within both the scientific team and staff is crucial when conducting research in diverse communities [65]. Inclusive hiring practices should be adopted to recruit researchers and staff members who reflect the populations being studied. By fostering a diverse team, the program can enhance cultural competency and better address the needs of various communities. Additionally, providing ongoing training and development on cultural competency, implicit bias, and community engagement will further equip researchers and staff to work effectively with diverse populations [4].

Improving data collection methods and survey design is another critical area. Surveys should be made shorter and more engaging, utilizing user-friendly digital interfaces and clear instructions to reduce participant fatigue and improve response rates. It is essential to expand the coverage of SDoH variables, including underexplored areas like gender identity, to gain a comprehensive understanding of factors influencing health outcomes [35]. Regular updates and validation of survey questions are necessary to maintain the relevance and accuracy of the data collected.

Mitigating selection bias and enhancing participation require targeted recruitment strategies focused on underrepresented groups. Outreach efforts in communities with low research participation rates, in collaboration with local organizations and community leaders, can help achieve this goal. Providing incentives for participation, such as financial compensation, childcare services, or transportation support, can address practical barriers and encourage broader participation [48].

Community engagement and empowerment are vital for the program’s success. Establishing community advisory boards with representatives from diverse communities can provide valuable insights and feedback on research priorities and methodologies, ensuring the research remains relevant and beneficial. Public education and awareness campaigns should be conducted to inform communities about the importance of research participation and its potential benefits. These campaigns should emphasize the program’s commitment to addressing health disparities and improving community health.

Fostering innovation and collaboration is key to the program’s advancement. Encouraging interdisciplinary collaboration among researchers, healthcare providers, and community organizations can foster innovation and enhance the impact of the research [66]. Collaborative efforts can lead to the development of holistic and comprehensive interventions that address multiple facets of health and well-being [67]. Implementing a continuous evaluation process to assess the effectiveness of strategies and make necessary adaptations ensures that the program remains responsive to the evolving needs of communities and maximizes its impact [68].

It is vital to provide personalized health programs using data from both individuals and communities. This involves educating the public about their role in contributing data and determining research priorities. Integrated healthcare systems should give priority to understanding the SDoH within the population, aiming to transition into learning health systems [32]. These systems actively gather data to improve clinical outcomes and boost the community. By emphasizing the role of community engagement, healthcare systems can better address the needs and challenges faced by diverse populations [69].

Furthermore, integrating mobile sensors and various technology platforms will enrich the completeness and value of the data for exploring hypotheses [70]. However, the expansion of wearable and sensor technologies must be balanced against privacy concerns and potential for increased surveillance, particularly for marginalized communities who have historically experienced disproportionate monitoring [71]. The program must ensure that adoption of these technologies includes robust consent processes, transparent data use policies, and participant control over data sharing [72]. Rather than gathering new datasets on individual travel and health behavior, researchers should creatively utilize existing data with advanced geocomputational tools while minimizing privacy intrusions. Geocomputation - a research field that applies computational technology and methods to geographic data [73], allows for the exploration of geospatial and Earth data using computational methods and tools [48]. This approach can provide a more nuanced understanding of how accessibility varies across space and time [74], while respecting participant privacy through aggregation and de-identification [75]. Currently, participants in the All of Us Program can choose to share their Fitbit data, and there are plans to include support for additional wearable devices in the future, with explicit opt-in mechanisms that give participants control [12]. The increasing use of wearable systems in health research indicates that wearable-driven studies may include underrepresented populations [76]. By integrating wearable systems with geospatial processing into the data tracking of the All of Us Program, researchers can broaden their investigation into underrepresented populations and their geographical locations while maintaining ethical safeguards against misuse [77]. This enables the exploration of innovative tracking methods, ultimately improving our understanding of the data to address disparities in these communities and expanding opportunities within the All of Us Program, provided that such expansion prioritizes participant autonomy and privacy protection.

## Conclusion

The All of Us Research Program aspires to represent a paradigm shift towards a more inclusive, equitable, and socially conscious approach to biomedical research. While the program has made important strides in participant diversity and data collection, translating these efforts into tangible community benefits and reduced health disparities remains an ongoing process that requires sustained commitment to addressing structural barriers and ethical challenges. By placing a strong emphasis on SDoH, the All of Us Research Program acknowledges the complexity of factors that influence health beyond traditional biomedical models. This recognition facilitates the development of more nuanced, effective public health interventions that can be tailored to the specific needs and challenges of different communities [7]. Moreover, the program’s dedication to addressing ELSI issues ensures that these advancements in healthcare and research are pursued with integrity, respect, and a deep sense of responsibility towards participant rights and privacy. As the program evolves, its commitment to empowering communities, advancing health equity, and fostering innovation in precision medicine continues to hold transformative potential for the future of healthcare.

## Electronic supplementary material

Below is the link to the electronic supplementary material.

Supplementary Material 1

## Data Availability

All data are incorporated into the article and its online supplementary material.
