# Supplementary material for: Returning value to communities from the All of Us Research Program through the lens of social determinants of health and ethical, legal, and social implications
Source: Int J Equity Health. 2026 Feb 2;25:58. doi: 10.1186/s12939-026-02758-6 (PMC12952018; doi:10.1186/s12939-026-02758-6)
Supplement: Supplementary file 1 — Supplementary Material 1 [file 12939_2026_2758_MOESM1_ESM.docx]

**Supplemental Table 1.** Search strategies for all databases

| **Database** | **Search String** |
| --- | --- |
| **PubMed/MEDLINE** | ("All of Us Research Program"[Title/Abstract] OR "All of Us Program"[Title/Abstract] OR "AoU"[Title/Abstract]) AND ("social determinants of health"[MeSH Terms] OR "SDoH"[Title/Abstract] OR "socioeconomic factors"[MeSH Terms] OR "economic stability"[Title/Abstract] OR "education access"[Title/Abstract] OR "healthcare access"[Title/Abstract] OR "neighborhood environment"[Title/Abstract] OR "social context"[Title/Abstract]) AND ("ethics"[MeSH Terms] OR "Ethical, Legal, and Social Implications"[Title/Abstract] OR "ELSI"[Title/Abstract] OR "bioethics"[MeSH Terms] OR "informed consent"[MeSH Terms] OR "privacy"[MeSH Terms] OR "data sharing"[Title/Abstract] OR "community engagement"[Title/Abstract] OR "health equity"[MeSH Terms] OR "underrepresented populations"[Title/Abstract] OR "diversity in research"[Title/Abstract]) |
| **EMBASE** | ('All of Us Research Program'/exp OR 'All of Us Research Program':ti,ab OR 'All of Us Program':ti,ab OR 'AoU':ti,ab) AND ('social determinants of health'/exp OR 'socioeconomic factors'/exp OR 'SDoH':ti,ab OR 'economic stability':ti,ab OR 'education quality':ti,ab OR 'healthcare access':ti,ab OR 'health care access':ti,ab OR 'neighborhood environment':ti,ab OR 'built environment':ti,ab OR 'social context':ti,ab) AND ('bioethics'/exp OR 'ethics'/exp OR 'Ethical Legal and Social Implications':ti,ab OR 'ELSI':ti,ab OR 'informed consent'/exp OR 'privacy'/exp OR 'data protection'/exp OR 'community engagement':ti,ab OR 'health equity'/exp OR 'minority health'/exp OR 'underrepresented populations':ti,ab OR 'diversity':ti,ab OR 'inclusion':ti,ab) |
| **Web of Science** | TS=("All of Us Research Program" OR "All of Us Program" OR "AoU") AND TS=("social determinants of health" OR "SDoH" OR "socioeconomic factors" OR "economic stability" OR "education access" OR "education quality" OR "healthcare access" OR "health care access" OR "neighborhood environment" OR "built environment" OR "social context" OR "community context") AND TS=("ethics" OR "Ethical Legal and Social Implications" OR "ELSI" OR "bioethics" OR "informed consent" OR "privacy" OR "data privacy" OR "data security" OR "data sharing" OR "community engagement" OR "participatory research" OR "health equity" OR "health disparities" OR "underrepresented populations" OR "minority populations" OR "diversity" OR "inclusion" OR "inclusivity") |
| **Cochrane Library** | ("All of Us Research Program" OR "All of Us Program"):ti,ab,kw AND ("social determinants of health" OR "SDoH" OR "socioeconomic factors" OR "economic stability" OR "education" OR "healthcare access" OR "neighborhood" OR "social context"):ti,ab,kw AND ("ethics" OR "ELSI" OR "Ethical Legal Social Implications" OR "bioethics" OR "privacy" OR "data sharing" OR "community engagement" OR "health equity" OR "underrepresented" OR "diversity"):ti,ab,kw |
| **Google Scholar** | "All of Us Research Program" OR "All of Us Program" AND ("social determinants of health" OR "SDoH") AND ("ELSI" OR "ethical legal social implications" OR "ethics" OR "community engagement" OR "health equity" OR "underrepresented populations") |
